# Factors Informing Outcomes for Older Cats and Dogs in Animal Shelters

**DOI:** 10.3390/ani8030036

**Published:** 2018-03-07

**Authors:** Sloane Hawes, Josephine Kerrigan, Kevin Morris

**Affiliations:** Institute for Human-Animal Connection, Graduate School of Social Work, University of Denver, Denver, CO 80208, USA; sloane.hawes@du.edu (S.H.); josephine.kerrigan@austinpetsalive.org (J.K.)

**Keywords:** companion animals, cat, dog, shelter, outcomes, euthanasia, geriatric, length of stay

## Abstract

**Simple Summary:**

Historically, older cats and dogs have been particularly at-risk for euthanasia in animal shelters due to their lower perceived appeal for adoption. This study found that the condition at intake had the greatest impact on the outcomes of older cats and dogs. Additionally, the application of specialized veterinary care, such as orthopedic surgery or chronic disease maintenance, is discussed as factors that inform higher rates of live outcomes for these senior companion animals. These findings demonstrate that if shelters integrate practices that address the specific needs of ageing companion animals, the live outcomes for this population can increase.

**Abstract:**

With advances in veterinary medicine that can increase the lifespan of cats and dogs and the effectiveness of spay/neuter programs in reducing the juvenile population of pets, animal shelters are experiencing an increasing population of older companion animals in their care. The purpose of this study was to assess the factors that inform the outcomes of these older cats and dogs. The sample consisted of 124 cats and 122 dogs that were over the age of 84 months (seven years) who were taken into a shelter over a one-year period. To assess the impact of condition at intake on the outcome for the senior animals, a multinomial logistic regression was performed. These findings indicate that preventative programming that can address the reasons these older animals are surrendered, as well as advancements in specialized medical or behavioral programs for ageing companion animals, may support an increase in live outcomes for older cats and dogs in shelters. Further study is needed to evaluate how the quality of life of older animals is impacted by remaining in the care of shelters rather than being euthanized.

## 1. Introduction

There is a growing body of evidence documenting the positive impacts of pet-keeping in communities. Particularly, the literature has demonstrated that efforts directed toward increasing pet-keeping through the adoptions of companion animals in shelters and rescues can result in improvements in public health [1,2,3,4]. These positive impacts on human health include increases in physical activity and cardiovascular health, use of social capital, maintenance of positive attachment relationships, and socioemotional health [5,6,7,8,9,10]. Despite the positive role that companion animals play in communities, there continue to be incidences in which families surrender their pets to animal shelters. The American Humane Association calculates that animal shelters across the United States take in an estimated 5–8 million cats and dogs every year [11]. The most commonly cited reasons for surrender include: aggressive behavior of the animal, housing-related issues, and caretaker’s personal issues [12]. This indicates that there continue to be limitations in the animal welfare safety net for some cats and dogs.

As spay/neuter of companion animals has become more widely practiced in the United States (U.S.), the population of companion animals in shelters has become less populated with juveniles and more populated with older cats and dogs [13,14,15]. Studies show there has been an increased incidence of surrender due to high medical costs [16]. Other studies have identified that animal illness and old age are the primary risk factors for being euthanized at the time of surrender [17]. Additional studies indicate that the chance of a dog or cat getting adopted significantly decrease with age of the animal due to high kennel competition against animals that have more “desirable” traits [18,19,20,21]. Furthermore, older animals are also more likely to be returned following adoption [22]. The result of these factors is that older cats and dogs are amongst those with the greatest risk for euthanasia in a shelter [23,24]. If lifesaving of sheltered companion animals is to continue to be optimized, then shelters should consider advancing their practices to support an ageing population.

Despite the evidence that age impacts the current outcomes for older cats and dogs in shelters, the literature has yet to document the influence of other factors in addition to age that can impact the outcomes of this population. This study evaluated the influence of a variety of factors on the outcomes of “senior” cats and dogs at the private, non-profit animal rescue, Austin Pets Alive! (APA), in Austin, Texas. Beyond age, this study also looked at other considerations for shelter decision-making, including breed, size, condition on intake, treatment plan, and reason for surrender. Understanding these variables and the relationship that they have to length of stay and live outcomes can support animal shelters in critically evaluating their policies and programs for older cats and dogs.

## 2. Materials and Methods

### 2.1. Data Collection

A retrospective cohort study was conducted on data obtained from APA’s ShelterLuv database. APA was selected due to its collection of innovative programs that are reported to result in a higher rate of live outcomes for animals who have been largely considered euthanasia candidates in traditional animal shelters [1]. As a private companion animal rescue, APA does not offer relinquishment services, but instead focuses on serving animals who are at-risk (for euthanasia) at Austin’s municipal shelter, Austin Animal Center (AAC, Austin, TX, USA), and other shelters and rescues in Texas. In 2016, APA took in over 7000 animals, many of which (39%) came as transfers from AAC due to medical or behavioral challenges. Data collected for the study included intake and outcome information for all cats and dogs that were 84 months or older and in the care of APA between 1 November 2016 and 24 November 2017. All of the animals included in the sample were admitted to AAC through either owner surrender or as strays. They were then transferred to APA from AAC after being flagged as “at risk” (for euthanasia) due to medical or behavior concerns. A total of 124 cats and 122 dogs over the age of 84 months fell within these selection criteria. While the definition of “senior” or “geriatric” for companion animals varies across species, breed, and size, 84 months was used as a generally recognized benchmark for the beginning of most aging-related medical or behavior symptoms, regardless of species or size [25,26]. Furthermore, 84 months is the criteria APA uses to determine eligibility for their discounted “senior” adoption fees.

Data collected for each animal in the study sample included date of intake to APA, intake type (e.g., owner surrender, stray, abandoned), estimated age at intake (in months), weight at intake, identified primary breed, reason for surrender to AAC (if applicable), qualitative description of condition upon intake at APA, qualitative description of plan for treatment once in the care of APA, total number of days in the custody of APA, the number of days while in the custody of APA when the animal was in off-site foster care, the number of days while in the custody of APA when the animal was on-site at APA, outcome date (if applicable), and outcome type (e.g., adoption, euthanasia, died in custody, still in care). The length of stay (time in custody, time on-site, and time in foster) for animals in the sample who had been returned to APA following an initial adoption were reported using the animals’ most recent time in APA’s care. The outcomes reported also reflect the animals’ status at the end of the study period on 24 November 2017. While not included within the APA data on the three length of stay variables (in custody, in foster, and on-site), the average time each animal was in the care of AAC prior to being transferred to APA was between 1–5 days.

Categories for reason for surrender to AAC included stray, people issues, animal illness, deceased caretaker, negligence, or abandoned. Stray was used if the animal was brought into AAC by an animal protection officer or by someone other than the animal’s caretaker. People issues encompassed reasons cited by the relinquishing family, such as: allergies, housing restrictions, and moving. Animal illness was used if the family of the senior cat or dog cited they could not afford to care for the animal anymore because the illness had become physically, economically, or emotionally difficult to sustain. Deceased caretaker was used if the animal was relinquished due to the death of one of the animal’s caretakers. Negligence was used if the animal was removed from a home by AAC due to a cruelty or neglect case. Abandoned was used if the animal had a microchip upon intake to AAC as a stray, but was never reclaimed by the individual listed on the microchip. Time in custody included the date the animal was accepted for transfer from AAC to the date the animal’s outcome at APA was recorded. For animals that are still in the care of APA, the final day of the study period, 24th November, was used as the last day for time in custody. Time in foster was the number of days of the animal’s total time in custody that was spent in off-site care with an APA registered foster family or in “pre-adopt” status in the home of a potential adopter. Time on-site was the number of days of the animal’s total time in custody that was spent on-site at APA in one of their kennels.

When possible, the data was coded into nominal or ordinal variables for the purposes of analyses. Identified primary breed was based upon what was indicated either by the relinquishing individual at intake (if applicable) or by the AAC staff member who conducted the animal’s initial evaluation. Breeds were then grouped according to the National Dog Show categories of: herding, hound, non-sporting, sporting, terrier, toy, or working [27]. The quantitative weight of dogs was coded into ordinal intervals of small (0–19 lbs), medium (20–59 lbs), large (60–99 lbs), and extra-large (100+ lbs). The qualitative descriptions of condition upon intake and plan for treatment were coded into nominal variables for analyses. Condition at intake descriptions, as determined by a veterinarian or veterinary technician, were coded into the following categories: terminal, poor body condition, further medical attention needed, and healthy. While most conditions were assessed at the time of the animals’ initial intake evaluation, some conditions, such as highly concerning behaviors or more complex medical diagnoses that required ongoing diagnostic tests, were amended in the animal’s case file once the final condition was determined. In this way, the coded condition upon intake descriptions reflect the final diagnosis of each animal following this thorough initial evaluation. Terminal condition for a medical reason included animals with confirmed cancer diagnosis, neoplasia, prolapsed rectum, renal failure in cats, heart failure, and/or poor mobility in dogs. Terminal condition for a behavior reason included animals that exhibited any of the three “unsafe” behavior categories: uninterruptible drive to fight, offensive aggression to humans, and unpredictable aggression. These behaviors were thoroughly assessed by the behavior management team who recorded incidents during dog-dog interactions or dog-human interactions. The documentation of these incidents informed further discussion by the Executive Director of APA, Director of Lifesaving Operations, and the Dog Behavior Manager, who would ultimately need to come to consensus on the terminal condition for a euthanasia decision to be confirmed. Poor body condition included animals who were underweight, had poor coat quality, skin issues, stiff joints, dehydration, poor teeth, and/or poor vision. Further medical attention needed included animals not eating, had nasal/ocular discharge, heart murmurs, enlarged masses that were not cancerous, and/or ulcers. Healthy condition included any other animals with no notable medical conditions aside from what could be reasonably expected due to their age. Animals with a healthy condition could have had behavior concerns at the time of transfer from AAC, but these behaviors were considered treatable within APA’s routine behavior program. Plan for treatment descriptions were coded into the following: monitor, exam needed, medication, and surgery. Animals coded as monitor were simply to be observed during their stay at APA, either because they required no additional medical intervention or because their medical condition was expected to be terminal and only veterinarian approval for euthanasia was required. Animals coded as monitor for behavior were to be watched until a later date as they showed no signs of aggression or dangerous behavior at the time of intake, but exhibited behaviors that may require additional intervention. Animals were monitored for behavior reasons to assess the intensity of a behavior concern, such as kennel aggression, swatting or hissing, areas of the body that are sensitive to touch, and resource guarding. Animals placed on medication were those experiencing pain, dehydration, or needed ongoing medication for a diagnosed medical condition. Those placed on medication for a behavior reason were for animals that experienced anxiety or fearfulness in the shelter setting that was prohibiting professionals to care for the animal at its highest capacity. The animals that were medicated for behavior concerns were considered a priority for foster. Animals with a treatment type of surgery required medical action for issues, such as mass removal, orthopedics, tooth extraction, or amputation. Animals who required an additional exam were those who needed to be further evaluated for medical or behavior concerns that could not be determined during their initial intake evaluation. These additional exam procedures included x-rays, blood tests (hematology and biochemical analysis), urinalysis, and/or other appropriate diagnostics provided by a commercial veterinary laboratory (e.g., Antech Diagnostics, Fountain Valley, CA, USA). Animals with behavior concerns requiring additional examination were given training opportunities to better assess the best placement option for the animal. These opportunities included the animal being enrolled in weekly socialization with other animals such as playgroups and/or receiving one-on-one obedience training such as the Canine Good Citizen Program to support the animal in developing qualities of a dependable and well-behaved pet. Following these additional exams, the animals were then placed into one of the other three treatment plan categories of monitor, medication, or surgery depending on their other needs, however this category was maintained for analysis insofar as it represents the group of animals that cannot be immediately determined as either an adoption or euthanasia candidate at the time of intake.

### 2.2. Statistical Analysis

A series of descriptive statistics were used to assess the sample of cats and dogs. A multinomial logistic regression was performed to determine the impact of the reason for surrender, condition at intake, and the treatment plan on the senior animals’ outcomes. A 95% confidence interval was used for all statistical analyses, with any result of a *p* value greater than or equal to 0.05 considered statistically significant.

## 3. Results

### 3.1. Descriptive Statistics

The sample of companion animals included in the study encompassed 124 cats and 122 dogs who were over the age of 84 months (7 years) and transferred from AAC to APA due to medical or behavior concerns. Most of the cats and dogs in the sample (55.5% and 55.8%, respectively) were 9 years or older (Table 1). A majority of the cats (81%) and dogs (55%) came to AAC as stray or abandoned (Table 2). Thirteen (10%) of the cats and 27 (22%) of the dogs were surrendered by their caretaker due to the animal’s medical concerns. Following the evaluation by AAC staff to verify the condition of the animals, 11 (9%) of the dogs and 22 (17%) of the cats were transferred from AAC due to behavior concerns, while 105 dogs (86%) and 101 cats (81%) were transferred due to medical needs. Six dogs (5%) and one cat (1%) were transferred on urgent medical status. A majority (72%) of the dogs weighed under 59 pounds (Table 3). A variety of breeds of both cats and dogs were present in the sample (Table 4). Beyond the factors included for analysis in the study (reason for surrender, condition at intake, treatment plan), it is notable that 35 of the dogs included in the study were heartworm positive, and three of the dogs had a bite case history. Within the sample of cats, five cats were ringworm positive, 14 cats were FIV positive, five cats were FeLV positive, and two cats were both FIV and FeLV positive. Five of the cats and nine of the dogs in the sample had been previously adopted from APA.

### 3.2. Length of Stay

**Figure 1:**
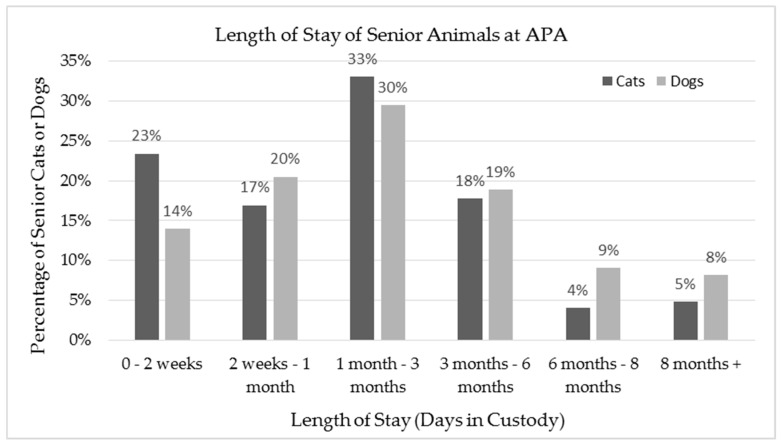
The total length of stay of the 124 cats and 122 dogs included in the sample, presented in percentage of all senior cats or senior dogs included in the sample.

The average time in custody for the cats included in the study (M = 68 days, SD = 73 days) was less than the average time in custody for dogs (M = 89 days, SD = 89 days) with length of stay that had a range of 1–348 days in custody for cats and 1–367 days in custody for dogs (Figure 1). The average time in custody for a random sample of 124 cats and 122 dogs that were in the care of APA over the same period, but not filtered for age over seven years, show that the senior cats and dogs have a longer average length of stay than the general population at APA (M = 57 days, SD = 48 days for general cat population; M = 51 days, SD = 64 for general dog population).

**Figure 2:**
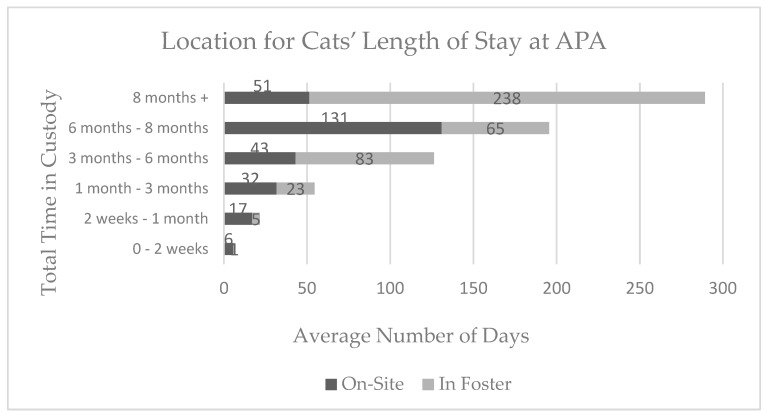
The average time spent in foster care versus on-site at Austin Pets Alive! (APA) for each range of total length of stay (time in custody) for the 124 cats in the sample.

**Figure 3:**
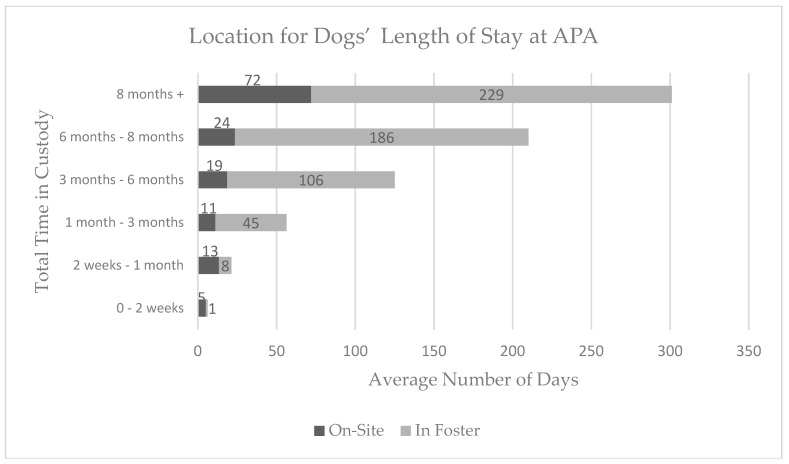
The average time spent in foster care versus on-site at APA for each range of total length of stay (time in custody) for the 122 dogs in the sample.

Throughout their length of stay, cats spent a greater amount of time on-site at APA (M = 30 days, SD = 37 days) than dogs (M = 18, SD = 32), with a range of 0–194 days spent on-site for cats and a range of 0–208 days spent on-site for dogs. Dogs spent a greater amount of time in foster homes (M = 71 days, SD = 82 days) than cats (M = 38 days, SD = 63 days), with a range of 0–367 days in foster for dogs and 0–345 days in foster for cats (Figure 2 and Figure 3).

### 3.3. Predictors of Outcomes

To determine the impact of condition at intake on the outcomes for the senior animals, a multinomial logistic regression was performed. To control for potentially confounding variables, reason for surrender was included along with condition at intake for the model. The outcomes for cats and dogs, grouped by their treatment plan are presented in Table 5. During exploratory data analysis, treatment plan was found to have a high degree of collinearity with assessment at intake, so it was excluded from the model for analysis. The relationship between size, particularly in dogs, and life expectancy has been well documented, therefore size was also excluded from the model [18,28,29,30,31,32,33]. Sample selection controlled for age of the animals.

Using the variables reason for surrender, condition at intake, and outcome of the senior animals, a significant model was achieved for cats (χ^2^(8) = 60.04, *p* < 0.01) and for dogs (χ^2^(8) = 62.61, *p* < 0.01).

According to the model generated based on this sample of senior cats, the cats who were surrendered due to animal illness and then assessed as a terminal condition were likely to be euthanized at a rate of 80% or adopted at a rate of 20%, while cats who were surrendered due to animal illness and then assessed as poor body condition or requiring further medical attention were likely to be adopted at rates of 99.5% and 98.3%, respectively. Cats that came to AAC as strays and were then assessed as a terminal condition were likely to be euthanized at a rate of 86.6% or died in the care of APA at a rate of 12%, while cats that came to AAC as strays and were then assessed as poor body condition or requiring further medical condition were likely to be adopted at rates of 72.8% and 56.8%, respectively, or likely to die in the care of APA at a rate of 22.7% and 30.4%, respectively.

Based on the model generated on this sample of senior dogs, dogs, regardless of reason for intake, were 100% likely to be euthanized if they were classified as terminal. Conversely, dogs who were assessed as poor body condition at intake were likely to be adopted, regardless of reason for surrender, at a rate of 100%. Dogs who were assessed as further medical attention needed, regardless of reason for intake were likely to be adopted at an average rate of 73.8% or to die in the care of APA at an average rate of 18.5%. Similarly, all healthy dogs, regardless of reason for intake, were likely to be adopted at an average rate of 71.5% or to die in the care of APA at an average rate of 14.3%.

These findings are largely consistent with the actual numbers found in the sample (Table 6). None of the senior cats or dogs included in the study sample were ultimately euthanized due to behavior reasons.

## 4. Discussion

Age has been a strong determinant of euthanasia decisions in animal shelters. Evidence suggests that animal welfare’s emphasis on advancing spay/neuter programs has contributed to progress in addressing the numbers of juvenile cats and dogs that are being surrendered to shelters [34,35,36]. However, these programs alone will not be effective in addressing the needs of the growing number of aging cats and dogs in shelters [37,38,39]. APA’s emphasis on providing medical and behavior resources to animals that may have previously been euthanized allows for an analysis of how increased populations of older cats and dogs may impact shelters’ lengths of stay and overall outcomes.

The results confirm that factors such as condition at intake influence the outcome of older cats and dogs at APA. Reason for surrender appeared to be a factor that was influencing outcomes for cats only. The significant impact of condition at intake suggests that there is a need for resources and programs that can support senior animals while they are still in their homes, either to reduce the overall number of senior animals relinquished or to improve the condition of animals that are ultimately surrendered. The observed collinearity between the treatment plan and condition at intake suggest that thorough medical and behavioral evaluations at intake can support shelter staff in more effectively addressing the needs of the animal while in their care. American Society of Anesthesiologists (ASA) status (an assessment of fitness of health before a surgical procedure) in veterinary medicine and the use of the marks developed from the Asilomar Accords in sheltering are similar systems for categorization that utilize information such as condition at intake to predict the outcome of the animal when in their care [40,41]. The usefulness of these systems is that they can support shelter staff in accounting for the resources that will need to go into the care of that animal and the ultimate risk that the animal will either die in their care or be euthanized. These findings support the use of systems like ASA or Asilomar, insofar as they are applied following thorough medical and behavioral evaluations and with consideration for the resources that are available to the shelter, rather than using the more basic metrics like age, coat color, or breed.

Senior animals may be euthanized in shelters because older cats and dogs often have longer lengths of stay that increases their risk of infectious disease [20]. Senior cats and dogs at APA were found to have a longer average length of stay than the general population that was cared for by APA over the study period. These findings are important insofar as numerous studies have documented the stress and health risk that result from increases in length of stay at shelters [12,42,43,44,45,46,47]. To date, there is no standardized definition in animal welfare for what specifically constitutes quality of life. While the average length of stay was 68 days for cats and 89 days for dogs in this study, most animals in the study (71% for cats and 87.7% for dogs) received some form of medical treatment during their time in APA’s custody. The relatively higher incidence of dying animals when compared to animals that were euthanized when they were assessed as requiring additional medical attention or having a poor body condition is representative of the live-outcome oriented culture of APA, rather than an inadequate assessment of these animals. Instead of using condition at intake as the determining factor, significant resources are allocated to all animals that are not believed to be terminal in an effort to support live outcomes for these animals. One study cited that cost is often a factor cited in euthanasia decisions [14]. Advancements in veterinary medicine to address chronic disease and illness now equip shelters with the ability to not only extend the lifespan of these senior companion animals but also ensure a higher quality of life through medication, vaccinations, and progressive surgical procedures than were previously possible [48]. Many of the more invasive medical procedures performed on the senior animals in this sample (e.g., dental extractions, mass removal, orthopedic surgery) were both costly and required extended periods of recovery or monitoring that likely contributed to these animals’ extended lengths of stay, but are anticipated to result in increased quality of life in the long-term. In this sample, 5.6% of cats and 23% of dogs received one of these higher cost surgical procedures. While further study is needed to weigh the costs of extended lengths of stay versus the benefits of these procedures on long-term quality of life, the data shows that if shelters are to effectively address the shift in population dynamics towards ageing companion animals, a corresponding shift in tolerance for an increased cost per animal may be needed.

The larger percentage of total time in custody that is spent in off-site foster care for these senior animals is an important finding in that placement in a foster home is believed to enhance the quality of life for animals who experience extended lengths of stay when in shelter custody. While the length of stay remains an important metric for assessing the potential for decreasing quality of life and/or non-live outcomes for sheltered animals, critically examining the additional factors such as resources allocated to these animals during that length of stay may support the goal of increasing live outcomes for this population, while also preserving the animal’s quality of life. Beyond documenting the overall length of stay and outcome from APA’s practices, assessing the physical and socioemotional impacts of this approach was beyond the scope of this study. Further studies are needed to examine how increasing the time and financial resources invested into veterinary or behavioral assessment of a senior animal on intake impacts both the shelter staff members’ and the animals’ experience in the shelter prior to their outcome.

While individual shelters may consider allocating their own resources to improved medical and behavioral assessment for all animals in their care, it is important to acknowledge that transfer partnerships and community partnering are emerging as effective approaches to increasing live outcomes for all at-risk animals in shelters, particularly when an individual organization’s resources are limited [26,49,50]. Integral to the effectiveness of AAC and APA in achieving live outcomes for the animals in their care is the partnership and resource-sharing that occurs between the two organizations and the personal investment that is demonstrated by the community through donations, fostering, and volunteer time [1]. Beyond increases in resources that are equipped to address the needs of senior animals, innovation in adoption programs may further optimize the potential for adoption of these senior animals. One study showed that, although older animals are less likely to be adopted, proactive programs at animal shelters that promote human-animal interaction and behavioral training can decrease the amount of time it takes for an animal to be adopted [19]. “Temporary adoption programs” that allow for the potential adopter to assess their suitability for meeting the animal’s need in their home prior to adoption have been shown to significantly reduce return rates [19]. Furthermore, programs that support families with senior animals prior to relinquishment should also be considered. The rising financial costs of veterinary care have been well-documented, with these effects being felt most acutely in communities of low socioeconomic status [51,52,53,54]. Additionally, families who provide homes for older cats and dogs may encounter compounding expenses as the animal ages and experiences additional medical or behavioral challenges. Further study is needed on the programs that are effectively addressing these gaps in communities.

## 5. Conclusions

By determining the gaps in shelters’ programs for older cats and dogs, shelter management can address the factors that have driven this population to be one of the most at-risk for euthanasia. Furthermore, this research highlights the importance of preventative outreach, specialized medical and behavior programs, and a strong foster care system that are equipped to address the needs of older animals in their homes and in a shelter.

## Figures and Tables

**Table 1:** Summary of age of cats and dogs included in the sample.

Age	Cat	Dog
7	26 (21%)	23 (18.9%)
8	28 (22.6%)	31 (25.4%)
9	9 (7.3%)	9 (7.4%)
10	19 (15.3%)	19 (15.6%)
11	6 (4.8%)	8 (6.6%)
12	12 (9.7%)	12 (9.8%)
13	6 (4.8%	8 (6.6%)
14	5 (4.0%)	6 (4.9%)
15	5 (4.0%)	3 (2.5%)
16	4 (3.2%)	1 (0.8%)
17	3 (2.4%)	1 (0.8%)
18	0 (0%)	0 (0%)
19	0 (0%)	1 (0.8%)

**Table 2:** Summary of reasons for intake at Austin Animal Center (AAC) for cats and dogs in the sample.

Reason of Intake	Cat	Dog
Stray	100 (80.6%)	58 (47.5%)
People Issues (allergic, relocating, etc.)	5 (4.0%)	16 (13.1%)
Deceased Caretaker	3 (2.4%)	1 (0.8%)
Negligence	2 (1.6%)	0 (0%)
Abandoned (chip and no reclaim)	0 (0%)	9 (7.4%)
Animal Illness	13 (10.5%)	27 (22.1%)
Behavior	1 (11.9%)	11 (9.0%)

**Table 3:** Summary of size of the dogs in the sample.

Size	Dog
Small (0–19 lbs)	38 (31%)
Medium (20–59 lbs)	50 (41%)
Large (60–99 lbs)	31 (26%)
X-Large (100+ lbs)	3 (2%)

**Table 4:** Summary of the breeds of cats and dogs in the sample.

Breed	Cat
Domestic Short hair	97 (78.2%)
Domestic Long hair	8 (6.5%)
Domestic Medium hair	9 (7.3%)
Manx	2 (1.6%)
Siamese	5 (4.0%)
Maine Coon	1 (0.8%)
Persian	1 (0.8%)
Russian Blue	1 (0.8%)
**Breed**	**Dog**
Sporting	23 (18.9%)
Hound	2 (1.6%)
Working	15 (12.3%)
Terrier	24 (19.7%)
Toy	29 (23.8%)
Non-Sporting	5 (4.1%)
Herding	23 (18.9%)

**Table 5:** Summary of actual numbers (*n* = 124 cats and 122 dogs) and percent of total sample of treatment plan.

Cat					
Treatment Plan	Euthanasia	Adoption	Died	Still in Care	Stolen/Lost
Exam	1 (0.8%)	3 (2.4%)	1 (0.8%)	3 (2.4%)	0 (0%)
Medication	19 (15.3%)	20 (16.1%)	9 (7.3%)	23 (18.5%)	0 (0%)
Surgery	0 (0%)	4 (3.2%)	2 (1.6%)	1 (0.8%)	0 (0%)
Monitor	3 (2.4%)	12 (9.7%)	2 (1.6%)	19 (15.3%)	2 (1.6%)
**Dog**					
**Treatment Plan**	**Euthanasia**	**Adoption**	**Died**	**Still in Care**	**Stolen/Lost**
Exam	7 (5.7%)	10 (8.2%)	0 (0%)	12 (9.8%)	0 (0%)
Medication	6 (4.9%)	20 (16.4%)	6 (4.9%)	18 (14.8%)	0 (0%)
Surgery	5 (4.1%)	15 (12.3%)	1 (0.8%)	7 (5.7%)	0 (0%)
Monitor	0 (0%)	5 (4.1%)	1 (0.8%)	9 (7.4%)	0 (0%)

**Table 6:** Summary of actual numbers (*n* = 124 cats and 122 dogs) and percent of total sample of condition at intake for cats and dogs.

Cat					
Condition	Euthanasia	Adoption	Died	Still in Care	Stolen/Lost
Terminal	19 (15.3%)	1 (0.1%)	2 (1.6%)	0 (0%)	0 (0%)
Healthy	0 (0%)	5 (4%)	0 (0%)	9 (7.2%)	1 (0.1%)
Poor body condition	1 (0.1%)	18 (14.5%)	5 (4%)	13 (10.5%)	1 (0.1%)
Further medical attention	3 (2.4%)	15 (12.1%)	7 (5.6%)	24 (19.4%)	0 (0%)
**Dog**					
**Condition**	**Euthanasia**	**Adoption**	**Died**	**Still in Care**	**Stolen/Lost**
Terminal	14 (11%)	0 (0%)	0 (0%)	0 (0%)	0 (0%)
Healthy	1 (0.1%)	5 (4.1%)	1 (0.1%)	2 (1.6%)	0 (0%)
Poor body condition	0 (0%)	17 (14%)	0 (0%)	12 (9.8%)	0 (0%)
Further medical attention	3 (2.5%)	28 (23%)	7 (5.7%)	32 (26.2%)	0 (0%)
